# Enzym

**Published:** 1899-12

**Authors:** 


### ENZYM.

Dr. Oscar Loew, a bacteriologist in the Agricultural Department of Washington, has published in a German medical journal a description of a newly discovered agent which he thinks, with other bacteriologists, may replace serum in the treatment of various forms of disease where that is now used. The new agent, which he says is similar to serum or antitoxin in some respects, and quite as potent, is manufactured without the aid of dumb beasts. Instead of introducing the deadly microbes into the blood of animals, and thus letting the disease-killing serum form, the noxious bacilli are confined in a glass jar, where they secrete a kind of ferment, which becomes so strong that it kills the very germs which make it, and even destroys their bodies. This fluid is the enzym, and is practically the same as antitoxin. In describing the new agent Dr. Loew says: "It has been known for a long time that certain bacterial growths in the animal body and in man will reach a point where they will cease, as of their own accord. In such cases the disease seems to run its full course, and then entirely die out of the system. According to previous theories this was due to acids, to noxious products or to the stimulation of the animal organism to itself produce a bacteria-killing product. It was never noted that in such cases the whole bacillus growth became completely dissolved by the time the course of the disease had been thus run. When I observed this, about a year and a half ago, I concluded that the germs which became thus dissolved had themselves produced the material which accomplished their dissolution. I communicated my theory to my friend, Dr. Rudolph Emmerich, bacteriologist, of Munich. We at one experimented upon a large scale, and produced enzymes.

"Our first enzym was produced by bacilli taken from ulcers formed in the disease known as pyocyaneus. A concentrated solution measuring one cubic centimetre killed, in from several hours to several days, many millions of bacilli of bubonic plague, cholera, typhoid fever, diphtheria, and anthrax. Thus you see the pyocyaneus bacilli formed an enzym fatal not only to themselves but to the germs of all these other diseases, and doubtless to many more which future work will determine.

"We next experimented upon animals. A large dose of bacilli causing anthrax, that dread disease fatal to brutes and often to man, was injected into a rabbit. The dose was sufficient to kill rapidly in from one to three days. Soon afterward from two to five cubic centimeters of the enzym solution were injected into the same animal. This treatment was repeated five or six times. More animals were similarly used, and all were cured of anthrax, and became healthy again. One or two were then painlessly killed and dissected.

"It was discovered upon microscopical examination that the anthrax bacilli had multiplied so rapidly in the beginning that they had reached as far as the liver and spleen. But the enzym injected into the blood had reached even these. It had destroyed and almost dissolved them. The enzym had thus acted in the animal body just as it had in the glass flask of the laboratory. Experiments with other disease germs showed results equally gratifying."—*Medical Times.*

